# Supplementary material for: Synergistic effect of probiotic, chicory root powder and coriander seed powder on growth performance, antioxidant activity and gut health of broiler chickens
Source: PLoS One. 2022 Jun 27;17(6):e0270231. doi: 10.1371/journal.pone.0270231 (PMC9236266; doi:10.1371/journal.pone.0270231)
Supplement: Attachment — Submitted filename: Response to reviewers.docx [file pone.0270231.s002.docx]

**Response to Reviewers**

**PONE-D-21-40040**

**Synergistic effect of probiotic, chicory root powder and coriander seed powder on growth performance, antioxidant activity and gut health of broiler chicken**

**Editorial comments**

| **comments** | **Reply by author** |
| --- | --- |
| 1. Please ensure that your manuscript meets PLOS ONE's style requirements, including those for file naming. | Followed the journal style |
| 2. We noticed you have some minor occurrence of overlapping text with the following previous publication(s), which needs to be addressed: | One of the publications was published by me, so there might be a chance of matching words as the methodology followed was the same. Duplicate reference in the reference section was identified and removed. |
| 3. We suggest you thoroughly copyedit your manuscript for language usage, spelling, and grammar. If you do not know anyone who can help you do this, you may wish to consider employing a professional scientific editing service. | Corrected the manuscript for language usage, spelling, and grammar. |
| 4. To comply with PLOS ONE submissions requirements, please provide methods of sacrifice in the Methods section of your manuscript. | Included in materials and methods |
| 5. In your Data Availability statement, you have not specified where the minimal data set underlying the results described in your manuscript can be found. PLOS defines a study's minimal data set as the underlying data used to reach the conclusions drawn in the manuscript and any additional data required to replicate the reported study findings in their entirety. All PLOS journals require that the minimal data set be made fully available. For more information about our data policy, please see http://journals.plos.org/plosone/s/data-availability. | Raw data was included |
| 6. . We note that you have stated that you will provide repository information for your data at acceptance. Should your manuscript be accepted for publication, we will hold it until you provide the relevant accession numbers or DOIs necessary to access your data. If you wish to make changes to your Data Availability statement, please describe these changes in your cover letter and we will update your Data Availability statement to reflect the information you provide. | Raw data was provided. Kindly update Data Availability statement. |

**Reviewer #1:**

| **Reviewer comments** | **Reply by author** |
| --- | --- |
| TITLE: Replace chicken with chickens. | **Modified as chickens** |
| Line 32: typo, please correct. Remove the extra “to” | **corrected** |
| Line 33: Please represent probiotic supplementation as percentage similar to chicory powder. | **Modified as per reviewer suggestion** |
| Line 37: IF you are representing antibiotic addition as gm/ton, please represent probiotic the same way. To avoid this confusion, I recommend representing your supplementations as percentage for all treatments. | **Expressed all additives in percentage** |
| Line 37: Please double check the abbreviation of gram. I believe it is “g”. | **Modified as ‘g’** |
| **Introduction:**  LINE 83: Remove the addition sign and replace with and. | **Removed typographical error** |
| **Material and methods:**  Line 108-113: Please include a table to describe the treatments clearly. | **Included** |
| Line 116: Are the total bacterial load 32 billion CFU/100 g or each probiotic was at this concentration? Is this a commercial product or designed by the authors? How did the authors check the viability of each bacteria in the probiotic cocktail? | **The probiotic used is a multistrain probiotic trail product produced by Intron Biologicals, Hyderabad and it contains a total of 10^9^ CFU/g of lyophilized and microencapsulated *Bacillus coagulans*, Saccharomyces boulardii, Lactobacillus acidophilus, Lactobacillus delbrueckii, Lactobacillus plantarum, Streptococcus thermophilus, Bacillus subtilis, Enterococcus faecium and Bifidobacterium bifidum. The viability was checked at the production itself.** |
| Line 120-122: Why are the authors presenting results in this section? I would recommend to move all the results presentation in the result section. | **Those lines were modified according to the materials and methods section. Removed lines 117 to 123.** |
| Line 118: Please represent probiotic levels as percentage too. | **Modified** |
| Line 130: “Feed intake”, please change to lower case F. | **corrected** |
| Line 134: Please include more details on how enzyme activity was determined. Describe how samples were stored, processed, followed by what type of assay was used. | ***Complete procedure of antoxidant enzyme activity has given below.** |
| Line 138: Please explain clearly how the 240 birds were sampled. It appears that only 1 bird/replicate that is 5 birds/treatment were processed for pH, intestinal contents etc. What about the other birds in the replicates? Why could the authors not process all birds for all samples to increase statistical power. | **One bird from each replicate means 8 birds for each treatment and total 48 samples were taken. According to the recommendations of IAEC, the number of birds to be sacrificed was limited as n=8 is enough for statistical analysis.** |
| Question: How many trials/studies were conducted with 240 birds (6 dietary treatments, 8 replicates)? | **Only one trial was conducted with 240 birds.** |
| Question: Where have the authors described the methodology for nutrient utilization? The results for nutrient utilization have been described in table 4 but I could not find the methodology portion for this section. | **We did not mentioned complete procedure of metabolic trial in methodology, however, we followed the standard procedure recommended by AOAC (1997).**  **The metabolic trial was conducted with one bird from each replicate to determine the retention efficiency of Dry Matter (DM), Crude Protein (CP) and energy as per the procedures described by AOAC (1997)** |
| Question: How did the authors selectively enrich for Salmonella from the samples? What enrichment procedure was employed to facilitate recovery of Salmonella? Did the authors conduct a Salmonella load test at the start of the trials before the treatments began? | Eight birds from each dietary treatment were slaughtered on 42^nd^ day and intestines were dissected at Meckel’s diverticulum. Approximately 5g of ileal digesta was collected aseptically into sterile sampling tubes and immediately transferred on ice to the laboratory for microbiological examination for *E. coli*, *Salmonella* spp and *Lactobacilli* spp counts. Salmonella-Shigella agar (SS Agar) for *Salmonella* spp. was used.  Then, 9 sterile test tubes with lids containing 9mL of phosphate buffer solution (PBS, pH-7.4) as diluent were prepared. Approximately 1g of the intestinal contents taken by sterile swab and homogenized for 3 minutes, aseptically mixed, added to the tubes, and diluted up to 10^9^. Later, 1ml of the contents of each test tube was transferred to one of three selective agar media on petri plates, respectively (Gunal *et al*., 2006). Aerobic bacterial plates ( *Salmonella* spp.) were placed in an incubator at 37^o^C for 24 hours. Finally, the intestinal bacterial colony populations formed in each plate was counted by colony counter and the number of colonies was expressed as log_10_ value.  **We did not conduct salmonella load test at the start of experiment as it was not required for our experiment.** |
| **Results and Discussion:**  Question: Why did the author not run groups for probiotic, chicory and coriander alone in the trials. Without these treatments, it is difficult to discern which component (probiotic, chicory, coriander) is effective in modulating body weight gains, FCR etc. | **Sir, we have conducted three separate experiments (other than this) with** probiotic @ 10 g, 15 g, and 20 gm per 100kg, coriander @ 0.5%, 1.0% and 1.5% & chicory root powder @ 0.5%, 1.0% and 1.5%. The results of above experiments clearly indicated that each group from probiotic, chicory root powder and coriander treatments had shown desirable performance at particular dosages. Probiotic at 10g/100 kg, chicory at 1.0 % and coriander at 1.5 % levels significantly (P<0.05) increased the overall performance of broilers when compared to control, antibiotic and other levels of respective treatments.  **The above experimental results/data was already been published in Plosone and Indian journal of animal sciences.**  I am sharing the link of above article for your reference.  https://doi.org/10.1371/journal.pone.0260923 |
| Table 7: Please share how the statistical analysis for comparing log CFU counts was conducted. I am interested to review log CFU/g values along with SE for each treatment. Some of the values for Lactobacillus (T5 7.88 vs T6 7.94), Salmonella (T4 7.33 vs T5 7.42) are very close to be statistically different from each other. | **** mentioned below** |

***ANTIOXIDANT RESPONSES**

The antioxidant enzymes such as glutathione Peroxidase (GSHPx), glutathione Reductase (GSHRx) and superoxide Dismutase (SOD) were estimated by following the methods of Paglia and Valentine (1967), Carlberg and Mannervik (1985) and Madesh and Balsubramanian (1998) respectively.

**3.8.1** **Glutathione Peroxidase (GSHPx) Enzyme Activity in Serum**

GSHPx activity was determined by the method proposed by Paglia and Valentine (1967) with slight modifications. Microtiter plates (96 well) were used to measure Glutathione peroxidase activity. To the 12.5 μl of serum, 250 μl of 0.1mM PBS (pH 7.4), 12.5 μl of H_2_O_2_ and 12.5 μl of reduced glutathione were added to wells and incubated at room temperature for 5 minutes, following which 12.5 μl of nicotinamide adenine dinucleotide phosphate (NADPH) solution was added and optical density was measured at 340 nm against the blank using ELISA reader - μQuant (BioTek instruments) for 5 minutes at 60 seconds interval and expressed as units/mg protein.

**Glutathione Reductase (GSHRx) Enzyme Activity in Serum**

GSHRx activity was determined according to method described by Carlberg and Mannervik (1985) with slight modifications. Microtiter plates (96 well) were used to measure Glutathione reductase activity. To the 12.5 μl of serum, 250 μl of 0.1mM PBS (pH 7.4), 12.5 μl of oxidized glutathione, 12.5 μl of FAD and12.5 μl of 80 mM EDTA were added and incubated at room temperature for 15 minutes. Optical density was measured at 340 nm against the blank by using ELISA reader - μQuant (BioTek instruments) for 5 times at 60 seconds interval after addition of 12.5 μl of NADPH solution at last and expressed as units/mg protein.

**Superoxide Dismutase (SOD) Enzyme Assay in Serum**

Microtiter plates (96 well) were used for assay of SOD activity. To the 100 μl of test sample, 6 μl of 1.25 mM 3-(4,5-Dimethylthiazol-2-yl)-2,5-Diphenyltetrazolium Bromide (MTT) was added in duplicate for each sample. 15 μl of 100 μM pyrogallol and 29 μl of 25 mM PBS were added to make the volume to 150 μl. Pyrogollol was freshly prepared and added after the addition of all other reagents and incubated for 10 minutes at room temperature and the reaction was terminated with addition of 150 μl of dimethyl sulfoxide (DMSO), which arrests the reaction and dissolves the MTT formazan crystals formed. Plates were shaken well and optical density recorded at 570 nm using ELISA reader - μQuant (Madesh & Balsubramanian, 1998).

****E. coli counts (**Calculated as per log_10_ colony forming units/gram of sample (10^6^).

| **Treatment** | **Replicates** | **colonies** | **cfu/ml (10^6^ dilution0** | **log 10 value** |
| --- | --- | --- | --- | --- |
| **T1** | **1** | **192** | **192000000** | **8.28** |
|  | **1** | **201** | **201000000** | **8.30** |
|  | **1** | **195** | **195000000** | **8.29** |
|  | **1** | **189** | **189000000** | **8.28** |
|  | **1** | **199** | **199000000** | **8.30** |
|  | **1** | **188** | **188000000** | **8.27** |
|  | **1** | **195** | **195000000** | **8.29** |
|  | **1** | **200** | **200000000** | **8.30** |
| **T2** | **2** | **12** | **12000000** | **7.08** |
|  | **2** | **14** | **14000000** | **7.15** |
|  | **2** | **12** | **12000000** | **7.08** |
|  | **2** | **15** | **15000000** | **7.18** |
|  | **2** | **14** | **14000000** | **7.15** |
|  | **2** | **15** | **15000000** | **7.18** |
|  | **2** | **16** | **16000000** | **7.20** |
|  | **2** | **16** | **16000000** | **7.20** |
| **T3** | **3** | **18** | **18000000** | **7.26** |
|  | **3** | **16** | **16000000** | **7.20** |
|  | **3** | **15** | **15000000** | **7.18** |
|  | **3** | **15** | **15000000** | **7.18** |
|  | **3** | **12** | **12000000** | **7.08** |
|  | **3** | **14** | **14000000** | **7.15** |
|  | **3** | **15** | **15000000** | **7.18** |
|  | **3** | **16** | **16000000** | **7.20** |
| **T4** | **4** | **16** | **16000000** | **7.20** |
|  | **4** | **19** | **19000000** | **7.28** |
|  | **4** | **18** | **18000000** | **7.26** |
|  | **4** | **25** | **25000000** | **7.40** |
|  | **4** | **22** | **22000000** | **7.34** |
|  | **4** | **23** | **23000000** | **7.36** |
|  | **4** | **24** | **24000000** | **7.38** |
|  | **4** | **25** | **25000000** | **7.40** |
| **T5** | **5** | **18** | **18000000** | **7.26** |
|  | **5** | **32** | **32000000** | **7.51** |
|  | **5** | **28** | **28000000** | **7.45** |
|  | **5** | **29** | **29000000** | **7.46** |
|  | **5** | **26** | **26000000** | **7.41** |
|  | **5** | **28** | **28000000** | **7.45** |
|  | **5** | **27** | **27000000** | **7.43** |
|  | **5** | **25** | **25000000** | **7.40** |
| **T6** | **6** | **16** | **16000000** | **7.20** |
|  | **6** | **14** | **14000000** | **7.15** |
|  | **6** | **15** | **15000000** | **7.18** |
|  | **6** | **16** | **16000000** | **7.20** |
|  | **6** | **18** | **18000000** | **7.26** |
|  | **6** | **13** | **13000000** | **7.11** |
|  | **6** | **17** | **17000000** | **7.23** |
|  | **6** | **16** | **16000000** | **7.20** |

**Reviewer #2:**

| **Reviewer comments** | **Reply by author** |
| --- | --- |
| **- Abstract**  Line 32-33 – please rephrase sentence as appropriate indicating the aim/background of the study. The details of the experimental design can be incorporated in the second sentence. | Included as per recommendations. |
| **Introduction**  Line76-77 – the connotation for antimicrobial properties of chicory root powder cannot be directly attributed but rather should be indicated as an indirect effect. Kindly rephrase the sentence appropriately. Or rather, if the authors had planned to indicate the antimicrobial activity other potential constituents, that may also be explained here. | Modified according to the recommendations |
| Line 84 – the information regarding postbiotics may be removed from here. The authors can alternately provide additional references that cite the effect of probiotics + inulin combinations on poultry gut health. Specifically, the authors may cite the strains of probiotics that have been used by other researchers in a concise manner. | Incorporated accordingly. |
| Line 90-92 – It would be better if the compounds listed in brackets are briefly expanded and described in terms of their chemical and functional properties/antimicrobial activities. | Sir, They were already proven compounds as indicated by reference, no need to discuss in detail. |
| Line 116 – Does this mean the concentration of each bacterial strain or is this the total lactic acid bacterial counts in general? | Total bacterial count (*Bacillus coagulans, saccharomyces boulardii, Lactobacillus acidophilus, Lactobacillus delbrueckii, Lactobacillus plantarum, Streptococcus thermophilus, Bacillus subtilis, Enterococcus faecium, Bifidobacterium bifidum)* |
| Line 116 and Table 1 – Isn’t this analysis termed Proximate Analysis? | Yes sir, proximate analysis |
| Line 118-121 – The way in which the treatment groups used for selecting the ideal formulation was not clear. What were the treatment groups involved in the broiler trials to determine the ideal composition of the mix that was eventually used for the final study? | **Sir, we have conducted three separate experiments (other than this) with** probiotic @ 10 g, 15 g, and 20 gm per 100kg, coriander @ 0.5%, 1.0% and 1.5% & chicory root powder @ 0.5%, 1.0% and 1.5%. The results of above experiments clearly indicated that each group from probiotic, chicory root powder and coriander treatments had shown desirable performance at particular dosages. Probiotic at 10g/100 kg, chicory at 1.0 % and coriander at 1.5 % levels significantly (P<0.05) increased the overall performance of broilers when compared to control, antibiotic and other levels of respective treatments.  **The above experimental results/data was already been published in Plosone and Indian journal of animal sciences.**  I am sharing the links of above articles for your reference.  https://doi.org/10.1371/journal.pone.0260923 |
| Line 132 – clearly specify the N value for the metabolic trial. | N value was already mentioned in table no 4.  The metabolic trial was conducted with one bird from each replicate means 8 birds treatment and total 48 samples were used for estimation of Dry Matter (DM), Crude Protein (CP) and energy |
| Line 155-170 – how were the Salmonella colonies confirmed just by observed the colony morphology from the SS agar. What if the colonies observed in the plates were from Proteus? | **Translucent colonies with black centers which are typical to Salmonella on SS agar were counted by using colony counter and the number of colonies was expressed as log_10_ value.**  The complete procedure was explained below.  Eight birds from each dietary treatment were slaughtered on 42^nd^ day and intestines were dissected at Meckel’s diverticulum. Approximately 5g of ileal digesta was collected aseptically into sterile sampling tubes and immediately transferred on ice to the laboratory for microbiological examination for *E. coli*, *Salmonella* spp and *Lactobacilli* spp counts. Eosin methylene blue agar (EMB) for *E. coli* growth, Salmonella-Shigella agar (SS Agar) for *Salmonella* spp. and MRS agar (De Man, Rogosa and Sharpe agar) for *Lactobacilli* spp growth was used.  Then, 9 sterile test tubes with lids containing 9mL of phosphate buffer solution (PBS, pH-7.4) as diluent were prepared. Approximately 1g of the intestinal contents taken by sterile swab and homogenized for 3 minutes, aseptically mixed, added to the tubes, and diluted up to 10^9^. Later, 1ml of the contents of each test tube was transferred to one of three selective agar media on petri plates, respectively (Gunal *et al*., 2006). Aerobic bacterial plates (*E. coli*, *Salmonella* spp.) were placed in an incubator at 37^o^C for 24 hours. Anaerobic (*Lactobacilli* spp.) medium plates were placed in an anaerobic jar with an anaerobic gas pack system at 37^o^C for 24 hours. Finally, the intestinal bacterial colony populations formed in each plate was counted by colony counter and the number of colonies was expressed as log_10_ value. |
| Line 171 – was a blinded histopathological evaluation and analysis performed for the respective tissue samples? | Yes sir, the HP slides were analyzed blindly, except the control group, so as to have a reference point. We have taken 8 histopathological sections per treatment each from each bird. |

The above manuscript has been modified as per reviewer suggestion
