# Supplementary material for: Synergistic effect of probiotic, chicory root powder and coriander seed powder on growth performance, antioxidant activity and gut health of broiler chickens
Source: PLoS One. 2022 Jun 27;17(6):e0270231. doi: 10.1371/journal.pone.0270231 (PMC9236266; doi:10.1371/journal.pone.0270231)
Supplement: Attachment — Submitted filename: Response to reviewers.docx [file pone.0270231.s003.docx]

**Response to editor comments**

**PONE-D-21-40040R2**

**Synergistic effect of probiotic, chicory root powder and coriander seed powder on growth performance, antioxidant activity and gut health of broiler chicken**

1. Please ensure that you refer to Table 1 in your text as, if accepted, production will need this reference to link the reader to the Table.

**Response:** Table 1 has been included in text and please provide reference link to Table 1.

2. Please amend the title either on the online submission form or in your manuscript so that they are identical.

**Response: corrected**

3. Please provide additional details regarding participant consent. In the Methods section, please ensure that you have specified (1) whether consent was informed and (2) what type you obtained (for instance, written or verbal). If your study included minors, state whether you obtained consent from parents or guardians. If the need for consent was waived by the ethics committee, please include this information.

**Response:** No consent was raised by animal ethics committee while obtaining permission. Moreover, the consent was not required for conducting experiments in Broilers. The same has been included in ethics statement.
